# Emergence and Genomic Characterization of a Hypervirulent *Klebsiella pneumoniae* Isolate From a New Clone in Brazil

**DOI:** 10.1111/1348-0421.70052

**Published:** 2026-03-27

**Authors:** Carlos Henrique Camargo, Ana Beatriz Nascimento Costa, Amanda Yaeko Yamada, Daniel de Sena Miranda, Pedro Smith Pereira Ferraro, Andreia Rodrigues de Souza, Amanda Maria de Jesus Bertani, Karoline Rodrigues Campos, Marlon Benedito Nascimento Santos, Rafaela Mastrangelo Rissetti, Carolina Lechinski de Paula, Patrik Júnior de Lima Paz, Maria Solange Duarte Soares, Christiane Salvador, Giovanna de Pinho Pieri, Jorge Siguemassa Higa, Daniela dos Santos Souza, Márcio Garcia Ribeiro, Claudio Tavares Sacchi, Monique Ribeiro Tiba‐Casas, Geraldine Madalosso, Denise Brandao de Assis

**Affiliations:** ^1^ Instituto Adolfo Lutz São Paulo Brazil; ^2^ Faculdade de Medicina Universidade de São Paulo São Paulo Brazil; ^3^ Faculdade de Saúde Pública Universidade de São Paulo São Paulo Brazil; ^4^ Department of Animal Production and Preventive Veterinary Medicine, School of Veterinary Medicine and Animal Science São Paulo State University‐UNESP Botucatu Brazil; ^5^ Laboratório Biomega Medicina Diagnóstica, Santo André São Paulo Brazil; ^6^ Divisão de Vigilância Epidemiológica, Secretaria da Saúde, São Bernardo do Campo São Paulo Brazil; ^7^ Divisão de Infecção Hospitalar, Centro de Vigilância Epidemiológica “Prof. Alexandre Vranjac” São Paulo Brazil

**Keywords:** community acquired infection, companion animals, Genomic surveillance, hypervirulent *Klebsiella pneumoniae*, MLST, public health

## Abstract

Hypervirulent *Klebsiella pneumoniae* (hvKp) has emerged globally as a major public health concern, often associated with severe community‐acquired and healthcare‐associated human infections. Despite alerts from PAHO/WHO on convergent hvKp strains with multidrug resistance in Latin America, comprehensive data from Brazil, the region's largest country, remain lacking. We performed a retrospective and prospective large‐scale surveillance of clinical, veterinary, and community isolates to identify hypervirulent and resistant *K. pneumoniae*. Among 1,008 isolates screened, one strain (ID_074_25) met hvKp criteria, displaying key virulence loci. Whole‐genome sequencing confirmed its genomic features, including a novel sequence type and a novel cluster in NCBI Pathogen Detection tool. This study underscores the need for national surveillance and capacity‐building in reference laboratories.

**Abbreviations**
ANI: average nucleotide identity
AST: antimicrobial susceptibility testing
CC: clonal complex
CG: clonal group
cKp: classical Klebsiella pneumoniae
CSF: cerebrospinal fluid
DLV: double‐locus variant
HAI: healthcare‐associated infection
hvKp: hypervirulent Klebsiella pneumoniae
IAL: Instituto Adolfo Lutz
ICEKp: integrative and conjugative element of Klebsiella pneumoniae
IMD: invasive meningococcal disease
IS: insertion sequence
MALDI‐TOF MS: matrix‐assisted laser desorption/ionization time‐of‐flight mass spectrometry
MGE: mobile genetic element
MIC: minimum inhibitory concentration
MLST: multilocus sequence typing
NCBI: National Center for Biotechnology Information
NHP: non‐human primate
PAHO: Pan American Health Organization
PCR: polymerase chain reaction
SLV: single‐locus variant
SNP: single‐nucleotide polymorphism
ST: sequence type
TSA: tryptic soy agar
wgMLST: whole‐genome multilocus sequence typing.
WGS: whole‐genome sequencing
WHO: World Health Organization

## 1 Introduction

*Klebsiella pneumoniae* is a major pathogen associated with both community‐acquired and healthcare‐associated human infections, ranging from urinary tract infections to severe pneumonia and sepsis [1]. Over the past decade, a distinct hypervirulent variant of *K. pneumoniae* (hvKp) has gained global attention due to its capacity to cause invasive infections, often in healthy individuals [2]. hvKp is distinct from classical‐acquired *K. pneumoniae* (cKp) in its ability to metastasize to distant sites, including most commonly the eye, lung and central nervous system [3].

Originating in East and Southeast Asia, hypervirulent *K. pneumoniae* (hvKp) strains have now been reported in distinct geographical regions. Early epidemiological and genomic studies from Asia demonstrated that invasive hvKp infections were predominantly associated with a limited number of clonal lineages, most notably capsular type K1 sequence type ST23 (clonal group CG23) and capsular type K2 lineages such as ST65 and ST86, with additional K2‐associated sequence types, including ST375, also reported among hvKp collections [4, 5, 6]. Population genomic analyzes further confirmed the early emergence and global dissemination of ST23 as a dominant hypervirulent lineage originating in Asia [7].

The classification of hvKp relies primarily on the detection of key virulence determinants, including the aerobactin synthesis locus *(iuc/iut*), salmochelin (*iro*), regulators of the mucoid phenotype (*rmpA/rmpA2*), and the *peg‐344* transporter, which are frequently carried on large virulence plasmids and are strongly associated with invasive disease [8]. Although hvKp strains were historically considered broadly susceptible to antimicrobial agents, recent reports from Asia have described the emergence of convergent hypervirulent and multidrug‐resistant lineages, particularly in China, where carbapenem‐resistant ST11 hvKp has been implicated in hospital outbreaks with high mortality [9].

The convergence of hypervirulence and antimicrobial resistance, particularly carbapenem resistance, poses a significant public health threat. Resistance to polymyxins (colistin/polymyxin B), once considered rare in hvKp, has increasingly been reported in Asian clinical settings, including colistin‐resistant hvKp isolates and within‐host evolution of polymyxin resistance during treatment, raising further concern regarding the convergence of virulence and last‐resort antimicrobial resistance [10, 11]. The Pan American Health Organization, a regional arm of World Health Organization, recently issued alerts concerning the emergence of such convergent strains in Latin America [12]. hvKp includes both chromosomal and plasmid‐encoded features that results in enhanced virulence through plasmid‐encoded transcriptional regulators *rmpA/rmpA2* (regulator of mucoid phenotype) controlling the chromosomal capsule (cps) loci and thereby causing the hypercapsule associated with the hypermucoviscous phenotype [3].

Despite this warning, the largest and most populous country in Latin America, Brazil, which has well‐documented healthcare inequalities and high rates of healthcare‐associated infections (HAIs) [13, 14] lacks comprehensive studies on hvKp. Therefore, this study primarily aims to provide a genomic alert on the emergence of a novel hvKp lineage in Brazil.

## 2 Methods

This is a retrospective study conducted at the Instituto Adolfo Lutz (IAL), the public health reference laboratory in the state of São Paulo, Brazil. The IAL routinely receives spontaneous submissions of bacterial isolates from healthcare‐associated infections for identification, antimicrobial susceptibility testing, resistance gene detection, and outbreak investigations. The *K. pneumoniae* isolates selected for this study included (i) human clinical isolates mostly recovered from hospitalized patients in public hospitals in the State of Sao Paulo, Brazil; (ii) isolates from veterinary sources (predominantly diseased companion animals and cattle); (iii) isolates of community‐acquired urinary tract infections (non‐hospitalized patients). All bacterial isolates were identified using MALDI‐TOF MS (Bruker Daltonics, Bremen, Germany) and tested for polymyxin B antimicrobial susceptibility via *in house* broth microdilution. String test was performed on fresh colonies grown on TSA to evaluate the hypermucoviscosity phenotype [15]. Resistance genes (*bla*_KPC_, *bla*_NDM_, *bla*_OXA‐48‐like_) [16, 17] and virulence loci (*rmpA*, *rmpA*2, *iucA*, *iroB*, *peg‐344*) [18] were assessed by PCR. A previous characterized isolate was employed as control [19]. hvKp was defined as an isolate with PCR‐based virulence markers plus a positive string test.

hvKp isolates underwent antimicrobial susceptibility testing (AST) and whole‐genome sequencing (WGS). Disk diffusion testing was performed according to Brazilian Committee on Antimicrobial Susceptibility Testing guidelines [20] for the following antimicrobial agents: aminoglycosides, including amikacin 30 µg, gentamicin 10 µg, and tobramycin 10 µg; penicillins and β‐lactam/β‐lactamase inhibitor combinations, such as amoxicillin‐clavulanic acid 30 µg, ampicillin 10 µg, and piperacillin‐tazobactam 36 µg; cephalosporins, comprising cefepime 30 µg (4th generation), cefotaxime 5 µg, ceftazidime 10 µg, and ceftriaxone 30 µg (3rd generation), as well as cefoxitin 30 µg (2nd generation) and the combination ceftazidime‐avibactam 14 µg; the monobactam aztreonam 30 µg; carbapenems, including imipenem 10 µg and meropenem 10 µg; fluoroquinolones, such as ciprofloxacin 5 µg and levofloxacin 5 µg; and the folate pathway inhibitor combination sulfamethoxazole‐trimethoprim 25 µg.

WGS was performed for species confirmation, resistance gene detection, and phylogenetic analysis. DNA was extracted using the Wizard Genomic DNA Purification Kit (Promega, Madison, USA), quantified with a Qubit fluorometer (Thermo Fisher Scientific, Waltham, USA), and sequenced using the Illumina DNA Prep Kit on a NextSeq platform (2 × 150 bp) (Illumina, San Diego, USA). For quality control, raw reads were checked using FastQC (https://github.com/s-andrews/FastQC) and Kraken2 was employed to identify potential contaminants [21], via Galaxy Europe platform [22]. Assembly was performed in CLC Genomics Workbench (Qiagen, Germantown, USA) and metrics were evaluated with QUAST [23]. Average nucleotide identity (ANI) was calculated using the JSpeciesWS webserver [24]. The generated fasta file was submitted to Kleborate platform (a genomic surveillance framework designed to analyze *K. pneumoniae* species complex (KpSC) genome assemblies) for sequence type (ST) determination and the detection of resistance and virulence genes [25]. Mobile genetic elements (MGE) were sought by Mobile Element Finder tool [26], an online tool available at Center for Genomic Epidemiology (https://www.genomicepidemiology.org/) webserver. To establish the clonal context of hvKp, raw reads were submitted to the NCBI Sequence Read Archive to be integrated in the NCBI Pathogen Detection Project. The assembled genome was automatically included in the *K. pneumoniae* SNP analysis workflow, which compared the isolate against a comprehensive database containing 132,095 genomes distributed across 11,908 SNP clusters (as of 16 July 2025). Comparisons were performed using a whole‐genome multi‐locus sequence typing (wgMLST) approach, with a clustering threshold of ≤ 25 allelic differences, as implemented by the NCBI Pathogen Detection pipeline. In addition, we conducted a phylogenetic reconstruction that included the single‐locus (SLV) and double‐locus variants (DLV) related to isolate ID_074_25 (SLVs: ST2039 and ST8224; DLVs: ST434, ST2056, ST3583, and ST6556), along with isolates previously characterized as hvKp from South America [27, 28]. A total of 23 genomes were retrieved and analyzed using Kleborate. The phylogeny of the 24 sequences was reconstructed with the CSI Phylogeny tool and visualized in Microreact [29].

## 3 Results

A total of 1008 *Klebsiella pneumoniae* complex isolates were analyzed, including 821 (81.5%) from human sources and 187 (18.5%) from animal origins, encompassing companion animals, livestock, and other veterinary sources (Supporting Information Figure S1). Antimicrobial susceptibility testing revealed a bimodal distribution of polymyxin B MIC values, with an overall MIC_50_ of 1 µg/mL and MIC_90_ 16 µg/mL. Human and non‐human isolates exhibited identical MIC_50_ values, whereas higher MIC_90_ values were observed among human‐derived isolates (Supporting Information Figure S2).

Screening for major carbapenemase genes demonstrated a high prevalence of *bla*_KPC_ and *bla*_NDM_ among human isolates, whereas these genes were rare or absent in non‐human sources (Table 1). No *bla*_OXA‐48_‐like genes were detected in the collection.

**Table 1:** Distribution of *K. pneumoniae* isolates by source and gene profile (*n* = 1,007).

Gene	Human (821)	Non‐human (187)	*p*‐value
*bla*_KPC_	556 (67.8%)	1 (0.5%)	0.0000
*bla*_NDM_	116 (14.1%)	0 (0.0%)	0.0000
*bla*_OXA‐48_	0 (0.0%)	0 (0.0%)	—
*rmpA*	1 (0.1%)	0 (0.0%)	1.0000
*iroB*	1 (0.1%)	2 (1.1%)	0.0902
*rmpA2*	0 (0.0%)	0 (0.0%)	—
*iucA*	2 (0.2%)	0 (0.0%)	1.0000
*peg‐344*	1 (0.1%)	0 (0.0%)	1.0000

In contrast to the widespread distribution of antimicrobial resistance markers, virulence‐associated genes classically linked to hypervirulent *K. pneumoniae* were exceedingly rare. The overall detection frequency *of rmpA, rmpA2, iroB, iucA*, and *peg‐344* was below 1% across the entire dataset (Table 1). Notably, only a single isolate fulfilled the combined molecular and phenotypic criteria for classification as hypervirulent *K. pneumoniae*. This isolate exhibited the full complement of virulence determinants and a positive string test, characterized by the formation of a viscous filament ≥ 5 mm.

This isolate, designated ID_074_25, was recovered from cerebrospinal fluid in the context of a severe community‐acquired infection with rapid clinical deterioration. The affected individual had underlying metabolic comorbidity and a history of multiple antimicrobial exposures in the preceding months. Empirical treatment with broad‐spectrum intravenous antimicrobials was promptly initiated; however, the clinical course was fulminant, progressing to a fatal outcome within 48 h of symptom onset. Microbiological investigation identified *Klebsiella pneumoniae* fully susceptible to all tested agents, including polymyxin B, and negative for common bacterial meningitis pathogens.

Genome of *K. pneumoniae* ID_074_25 consisted of 63 contigs (average coverage of approximately 104X), with a total length of 5.4 Mb and N50 of 281,956 bp; GC content was estimated at 57.2%. Average nucleotide identity (ANI) was calculated showed > 99.9% identity with reference *K. pneumoniae* strains (Supporting Information Table S2). It harbored only intrinsic resistance determinants, including *bla*_SHV‐11_, *fosA*, *oqxAB*, and a point mutation in *ompK*35 (E132K). The analysis of MGE confirmed the presence of a limited resistance repertoire genes consisting mainly of *bla*_SHV_ variants, *fosA*, and *oqxAB*, all located on chromosomal contigs with no evidence of association with mobile genetic elements carrying resistance genes. The MGE analysis identified multiple insertion sequences (IS*Sty2*, IS*Ecl10*, IS*Kpn2*, IS*Eam1*, and IS*Sen4*); however, none were located in proximity to the virulence loci. Plasmid replicons IncFIB(K) and IncFII were identified, but no plasmid‐borne translocatable units carrying virulence genes were detected.

Virulence genotypes consistent with hypervirulent lineages were identified, including multiple siderophore systems, such as yersiniabactin (*ybt12*; ICEKp1‐374‐1LV), colibactin (*clb1*; 67‐1LV), aerobactin (*iuc2*), and salmochelin (*iro2*). Additionally, the isolate carried the *rmp2* gene associated with the KpVP‐2 virulence plasmid, though *rmpA2* was not detected. *clbB/N*, *iroBCDEN*, *iucABCD*, *peg‐344*, *rmpA/C*, and *ybtPQ* were also detected. Capsular type KL2 O1/O2v1 was identified. The MLST analysis identified the hvKp ID_074_25 as a novel sequence type (ST8224) with allelic profile *gapA* 427, *infB* 3, *mdh* 2, *pgi* 4, *phoE* 9, *rpoB* 4, *tonB* 13. Plasmid replicon analysis identified the presence of IncFIB(K) with 99.1% identity and 100% coverage (Genbank Accession Number JN233704).

The genome of hvKp ID_074_25 was found to be genetically distinct from all existing SNP clusters in the NCBI Pathogen Detection database. Specifically, no matches were identified within the 25‐allele threshold in wgMLST comparisons, indicating that the isolate does not cluster with any known *K. pneumoniae* clones currently in the database. On the other hand, a more relaxed analysis with cutoff of up to 1000‐allele threshold showed that hvKp ID_074_25 target was related only with four genomes (all of them belonging to ST2039, of which ST8224 is a single locus variant – SLV) for which different alleles ranged from 914 to 999 (by using the NCBI Pathogen Detection platform): PDT001011263.1 (2015, Norway, feces): 4,396 shared alleles; 914 different alleles; PDT002383263.1 (2015, Norway, feces): 4,392 shared; 924 different; PDT002714871.1 (2020, Colombia, respiratory sample): 4,369 shared; 940 different; PDT001494582.1 (2020, Lithuania, aspirate): 4,354 shared; 999 different. All five isolates, including ID_074_25, shared an identical set of hypervirulence‐associated genes, including yersiniabactin (*ybtP, ybtQ*), colibactin (*clbB, clbN*), aerobactin (*iucA, iucB, iucC, iutA*), salmochelin (*iroB, iroC, iroD, iroN*), as well as regulators of mucoid phenotype (*rmpA, rmpC*) and the *peg*‐344 transporter.

**Figure 1:**
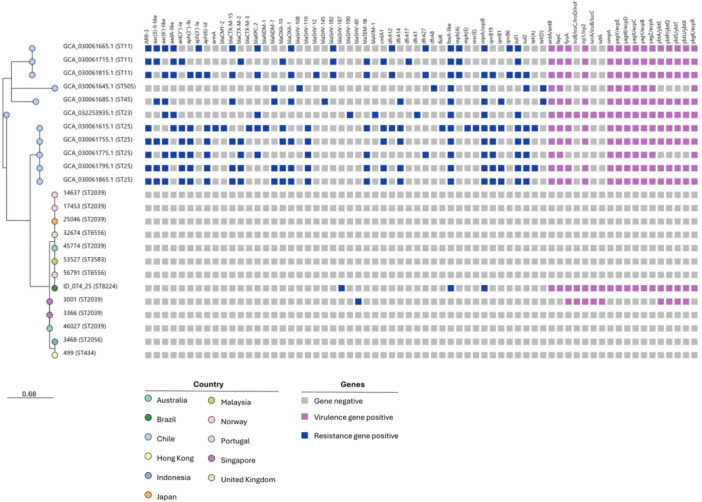
Phylogenetic reconstruction of *Klebsiella pneumoniae* isolates related to ID_074_25 (deep green circle). Colored circles at the branch tips indicate the countries of origin, while blue and purple squares represent resistance and virulence genes, respectively. Sequence types (STs) are shown in parentheses next to each isolate sequence.

The second phylogenetic analysis revealed that isolate ID_074_25 (ST8224) clustered within a clade composed of distinct sequence types from diverse global regions, reflecting evolutionary relatedness rather than shared virulence or resistance traits (Figure 1). While most neighboring STs lack classical hypervirulence determinants or acquired resistance genes, ID_074_25 uniquely harbors multiple virulence‐associated genes. Compared with South American isolates (particularly those from Chile) it shows greater phylogenomic divergence: Chilean strains display broader antimicrobial resistance repertoires typical of multidrug‐resistant lineages, whereas ID_074_25 retains a limited resistance profile but a virulence gene complement similar to its regional counterparts. This genomic configuration may represent an early signal of a lineage with reduced resistance burden yet heightened virulence potential, underscoring concern for future convergence toward hypervirulent–resistant phenotypes.

This Whole Genome Shotgun project has been deposited at DDBJ/ENA/GenBank under the accession JBPQHI000000000. The version described in this paper is version JBPQHI010000000. The genome was also deposited to the Pasteur MLST database under ID76146.

## 4 Discussion

The identification of a hypervirulent *K. pneumoniae* (hvKp) strain in Brazil, designated ID_074_25, marks a critical finding in the context of national genomic surveillance. Recovered from a fatal case of community bacterial meningitis, this strain harbored a full constellation of virulence determinants, including yersiniabactin, colibactin, aerobactin, salmochelin, *peg‐344*, and *rmpA/C*, and was assigned to capsular type KL2, with complete in vitro susceptibility to all tested antimicrobials, including polymyxin B.

Notably, the patient was a previously healthy postal worker with no recent hospitalization, who developed meningitis following a seemingly benign episode of non‐suppurative otitis media. Despite prompt empirical treatment and intensive supportive care, the clinical course was fulminant and resulted in death within 48 h. This outcome underscores the intrinsic pathogenic potential of hvKp and reinforces its ability to cause severe, life‐threatening infections outside the healthcare setting, even in the absence of antimicrobial resistance.

Genomic analysis identified the isolate as a novel sequence type (ST8224), a single‐locus variant of ST2039. Comparative evaluation against more than 11,000 SNP‐defined clusters in the NCBI Pathogen Detection database revealed no close matches, even under relaxed thresholds, suggesting that ID_074_25 represents a previously unrecognized genomic lineage. The relationship to ST2039, whose publicly available genomes are all hypervirulent and human‐derived, points to the emergence of a new hvKp sublineage with epidemiological significance in Brazil. This finding was supported by a second phylogenetic analysis including the single‐locus (SLV) and double‐locus variants (DLV) of ST8224. The results confirmed the uniqueness of this clone, occupying a phylogenetic position distinct from well‐characterized hypervirulent *K. pneumoniae* (hvKp) lineages such as ST23, ST65, and ST86 [8]. In contrast, Chilean isolates carried both virulence and resistance determinants, further highlighting their distinct genomic profiles compared to ID_074_25.

Similar scenarios have been documented globally. In China, outbreaks of carbapenem‐ and colistin‐resistant hvKp have been reported [9, 30, 31], while in the United States, community‐acquired hvKp infections have been increasingly recognized [32, 33]. These reports, along with our findings, challenge the historical view of hvKp as a low‐risk, hospital‐bound pathotype.

In Brazil, genomic surveillance of hvKp is still incipient. Most available data stem from hospital‐based isolates, with limited insight into community circulation or restricted to case reports. One exception is the report of an epizootic outbreak that killed 11 captive marmosets in São Paulo, caused by a fully susceptible hvKp ST86 strain [19]. In Santa Catarina, a state from South Brazil, described the first short outbreak in NHP (non‐human primate) caused by *K. pneumoniae* displaying a hypermucoviscosity phenotype and belonging to capsular serotypes K1 and ST23, with the presence of the *magA* and *rmpA* genes [34]. Although genetically unrelated to ID_074_25, this case highlights the pathogen's potential for multispecies transmission and ecological adaptation, underscoring the relevance of a One Health framework [12, 35].

While hypervirulence determinants were rare across the broader collection of 100 *K. pneumoniae* isolates analyzed in this study, the detection of a genomically divergent hvKp strain in a fatal case from the community carries substantial implications. Brazil faces high rates of antimicrobial resistance, often driven by mobile genetic elements [36, 37, 38]. The higher prevalence of KPC and NDM in clinical isolates was expected, as isolates sent to our reference laboratory are typically pre‐screened by local laboratories, resulting in a sampling bias. In this setting, the convergence of resistance and virulence, already documented in other countries, could evolve into a major public health crisis. The structural disparities in healthcare access, antibiotic use, and surveillance capacity across Brazilian regions further exacerbate this risk.

Although this study is strengthened by a large and diverse dataset, the lack of more detailed epidemiological metadata for most human and non‐human isolate, and the absence of long reads to determine the location (chromosome or plasmid) of virulence genes in ID_074_25, restrict some conclusions, which may be considered limitations of the current study. Another limitation that must be stated is that while the presence of virulence genes and the positive string test are suggestive, no functional assays (e.g., *Galleria mellonella* or other infection model, or serum resistance) were performed to confirm the hypervirulent phenotype. We emphasize that the present work was not intended to provide phenotypic validation of virulence, but rather constitutes a surveillance‐oriented genomic report of a novel hypervirulent *K. pneumoniae* lineage (ST8224), revealed by large‐scale screening efforts and associated with a fatal community‐acquired infection in Brazil. Strengthening national genomic surveillance efforts and integrating them with clinical and epidemiological networks is essential to bridge this gap.

## 5 Conclusion

This large‐scale study documents the genomic identification of a novel hypervirulent *Klebsiella pneumoniae* lineage in Brazil among more than a thousand of isolates. Far from being an isolated finding, a potential shift in the local epidemiology of *K. pneumoniae*, with serious implications for public health and antimicrobial stewardship can be speculated. Furthermore, it reflects the silent evolution of high‐risk clones in regions where genomic surveillance remains limited. In settings marked by high antimicrobial resistance, fragmented health systems, and socioeconomic inequities, the convergence of virulence and resistance is a foreseeable reality. Sustained investment in integrated, cross‐sectoral genomic surveillance is critical to anticipating and mitigating the clinical and epidemiological impact of emerging virulent and resistant *K. pneumoniae* lineages.

## Author Contributions

**C.H.C.:** conceptualization, resources, investigation, writing – original draft, writing – review and editing. **A.B.N.C., A.Y.Y., D.S.M., P.S.P.F., A.R.S., A.M.J.B., K.R.C., M.B.N.S., R.M.R., C.L.P., P.J.L.P.:** laboratory. **M.S.D.S., C.S., G.P.P., J.S.H., D.S.S.:** investigation. **M.G.R.:** conceptualization, resources, writing – review and editing. **C.T.S., M.R.T.C.:** laboratory, writing – original draft. **G.M., D.B.A.:** conceptualization, investigation, writing – review and editing.

## Disclosure

During the preparation of this work the authors used ChatGPT 5.0 in order to checking grammar, spelling, and text fluidity. After using this tool, the authors reviewed and edited the content as needed and take full responsibility for the content of the publication.

This Whole Genome Shotgun project has been deposited at DDBJ/ENA/GenBank under the accession JBPQHI000000000. The version described in this paper is version JBPQHI010000000.

## Ethics Statement

The study was registered with the Technical‐Scientific Council (Conselho Técnico‐Científico, CTC‐IAL 04‐Q/2024) of the Adolfo Lutz Institute and was exempt from ethics review, as it did not involve any intervention with humans and did not directly involve human participants.

## Conflicts of Interest

The authors declare no conflicts of interest.

## Supporting information


**Figure S1:** Sunburst chart illustrating the hierarchical distribution of *Klebsiella pneumoniae* isolates (*n* = 1008) according to their source of origin.

**Figure S2:** Distribution of minimum inhibitory concentrations (MICs) of polymyxin B among *Klebsiella pneumoniae* isolates (*n* = 1008), stratified by source.

**Table S1:** Results of polymyxin B and PCR for carbapenemases and virulence genes for *K. pneumoniae* complex isolates (*n* = 1008).

**Table S2:** ANI Results for *K. pneumoniae* isolate ID_074_25.

**Table S3:** Data of *Klebsiella pneumoniae* genomes used in the phylogenetic construction.

## Data Availability

Whole‐genome sequencing data generated in this study have been deposited in the GenBank database under the accession numbers listed in the Supplementary Tables. All relevant phenotypic, epidemiological, and genomic data supporting the findings of this study are provided in the main text and Supporting Materials. Additional data are available from the corresponding author upon reasonable request.
