# Supplementary material for: Emergence and Genomic Characterization of a Hypervirulent Klebsiella pneumoniae Isolate From a New Clone in Brazil
Source: Microbiol Immunol. 2026 Mar 27;70(5):261–7. doi: 10.1111/1348-0421.70052 (PMC13140776; doi:10.1111/1348-0421.70052)
Supplement: Supplementary file 1 — Figure S1: Sunburst chart illustrating the hierarchical distribution of Klebsiella pneumoniae isolates (n = 1008) according to their source of origin. [file MIM-70-261-s003.pdf]

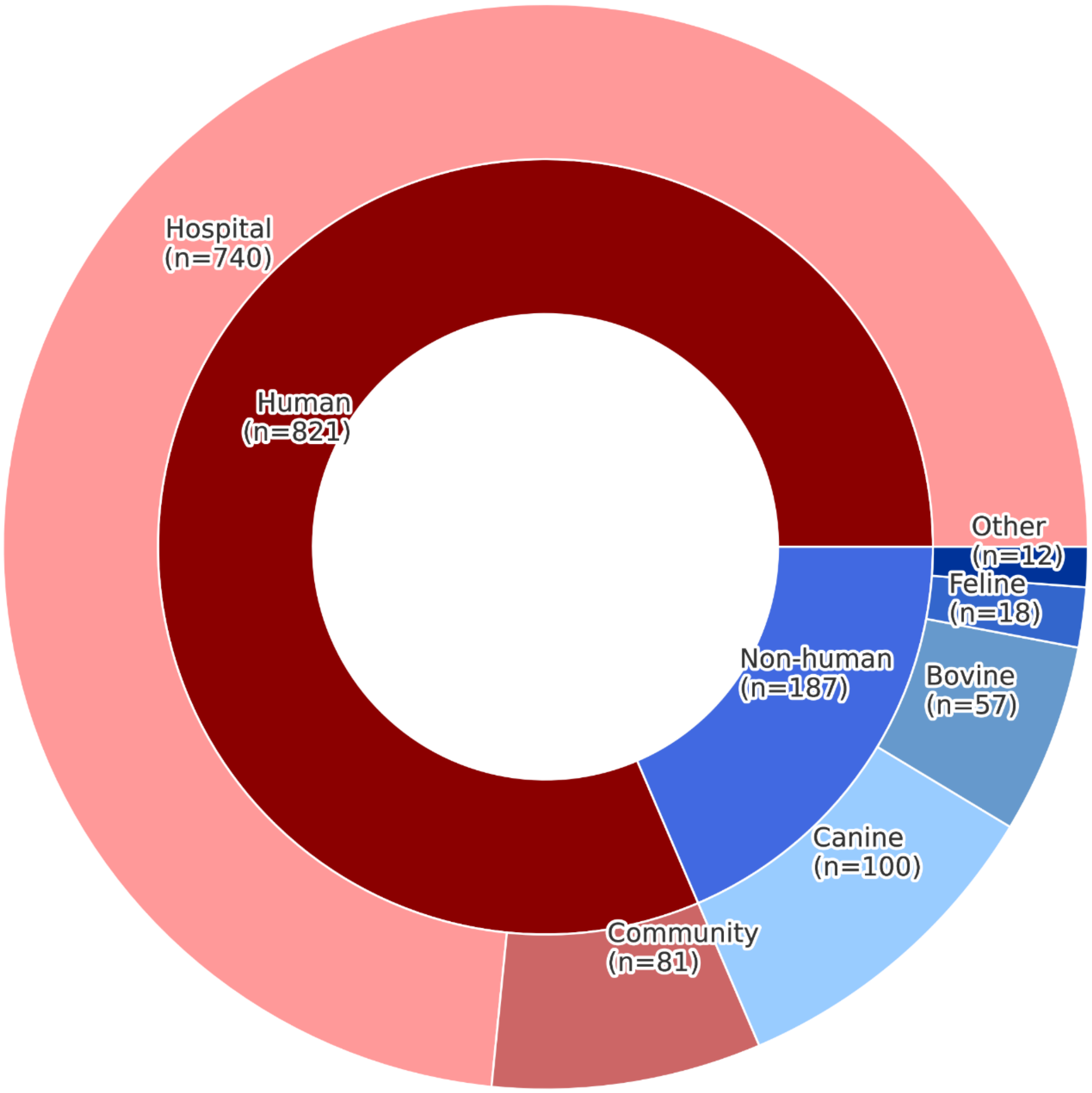

Figure S1. Sunburst chart illustrating the hierarchical distribution of *Klebsiella pneumoniae* isolates (n = 1008) according to their source of origin. The inner ring categorizes isolates as of human or non-human origin, whereas the outer ring subdivides human isolates into hospital-associated and community-acquired, and non-human isolates into bovine, canine, feline, and other sources. Segment size is proportional to the number of isolates within each category, with absolute counts displayed. Color scheme reflects origin type, using red gradients for human-derived isolates and blue gradients for non-human-derived isolates.
