# Supplementary material for: Emergence and Genomic Characterization of a Hypervirulent Klebsiella pneumoniae Isolate From a New Clone in Brazil
Source: Microbiol Immunol. 2026 Mar 27;70(5):261–7. doi: 10.1111/1348-0421.70052 (PMC13140776; doi:10.1111/1348-0421.70052)
Supplement: Supplementary file 2 — Figure S2: Distribution of minimum inhibitory concentrations (MICs) of polymyxin B among Klebsiella pneumoniae isolates (n = 1008), stratified by source. [file MIM-70-261-s002.pdf]

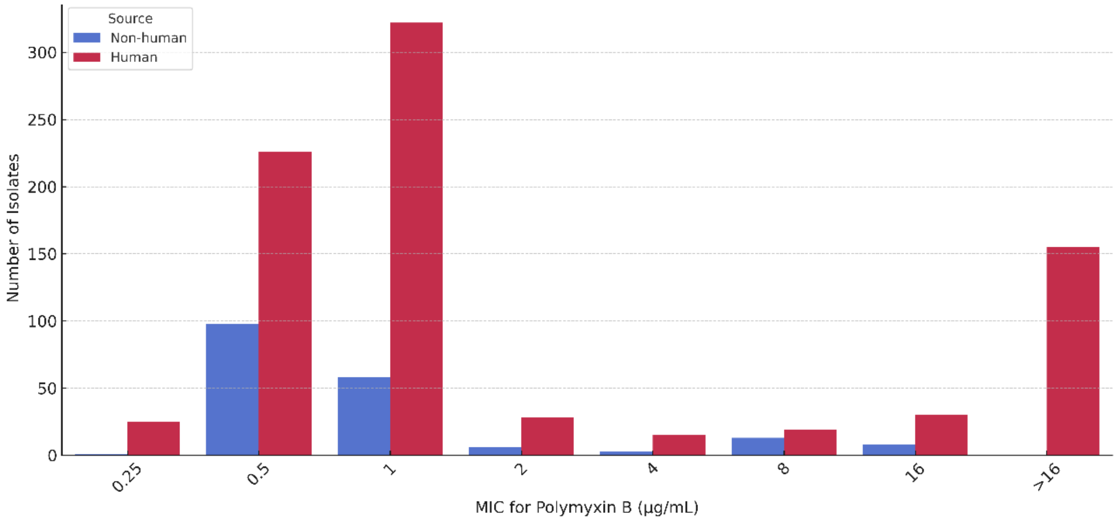

Figure S2. Distribution of minimum inhibitory concentrations (MICs) of polymyxin B among *Klebsiella pneumoniae* isolates (n = 1008), stratified by source. The x-axis depicts MIC values (µg/mL), and the y-axis represents the number of isolates per MIC category. Human isolates are highlighted in red, whereas non-human isolates are shown in blue, demonstrating distinct MIC profiles across ecological origins and enabling comparison of susceptibility trends between clinical and environmental reservoirs.
